# Immortalized tumor derived rat fibroblasts as feeder cells facilitate the cultivation of male embryonic stem cells from the rat strain WKY/Ztm

**DOI:** 10.1186/2193-1801-3-588

**Published:** 2014-10-08

**Authors:** Nils-Holger Zschemisch, Regina Eisenblätter, Cornelia Rudolph, Silke Glage, Martina Dorsch

**Affiliations:** Institute for Laboratory Animal Science and Central Animal Facility, Hannover Medical School, Carl-Neuberg-Str.1, 30625 Hannover, Germany; Institute for Molecular and Cellular Pathology, Hannover Medical School, Carl-Neuberg-Str.1, 30625 Hannover, Germany

**Keywords:** Tumor derived rat fibroblasts, ES cells, Chimera, Germ line transmission, Feeder cells, Gender dependency

## Abstract

Feeder cells are essential for the establishment and culture of pluripotent rat embryonic stem cells (ESC) *in vitro*. Therefore, we tested several fibroblast and epithelial cell lines derived from the female genital tract as feeder cells to further improve ESC culture conditions. The immortalized tumor derived rat fibroblast TRF-O3 cells isolated from a *Dnd1*-deficient teratoma were identified as optimal feeder cells supporting stemness and proliferation of rat ESC. The TRF-O3 cells were characterized as myofibroblasts by expression of fibroblast specific genes *alpha-2 type I collagen, collagen prolyl 4-hydroxylase alpha (II), vimentin, S100A4, and smooth muscle* α-actin. Culture of inner cell masses (ICM) derived from WKY/Ztm rat blastocysts in 2i-LIF medium on TRF-O3 feeder cells lacking *LIF, SCF* and *FGF2* expression resulted in pluripotent and germ-line competent rat ESC lines. Therein, genotyping confirmed up to 26% male ESC lines. On the other hand the TRF-O3 specific *BMP4* expression was correlated with transcriptional activity of the mesodermal marker *T-brachyury* and the ectoderm specific *nestin* in the ESC line ES21 demonstrating mesodermal or ectodermal cell lineage differentiation processes within the ESC population. Substitution of 2i-LIF by serum-containing YPAC medium supplemented with TGF-β and rho kinase inhibitors or by 4i medium in combination with TRF-O3 feeder cells led to enhanced differentiation of ES21 cells and freshly isolated ICMs. These results suggest that the ESC culture conditions using TRF-O3 feeder cells and 2i-LIF medium supported the establishment of male ESC lines from WKY/Ztm rats, which represent a favored, permissive genetic background for rat ESC culture.

**Electronic supplementary material:**

The online version of this article (doi:10.1186/2193-1801-3-588) contains supplementary material, which is available to authorized users.

## Introduction

Rat ESC lines are a tool for gene targeting (Tong et al. 2011; Yamamoto et al. 2011), the generation of transgenic animals (Kawamata and Ochiya 2010, Kawamata and Ochiya 2011), and for *in vitro* differentiation approaches (Wang et al. 2012; Peng et al. 2013). In 2008 the first pluripotent rat ESC lines derived from blastocysts of inbred Dark Agouti (DA) and outbreed Spargue-Dawley (SD) rats using 2i-LIF medium (Buehr et al. 2008; Li et al. 2008) were established. Thereafter, several ESC lines were cultured from ICMs of Wistar, Long-Evans, and SHR rat blastocysts. (Li et al. 2009a; Zhao et al. 2010; Blair et al. 2011a; Fernandez et al. 2011; Tong et al. 2011; Yamamoto et al. 2011; Hong et al. 2012). Unexpectedly, the ESC lines emerged from these experiments showed predominantly a female genotype (Blair et al. 2011b).

Besides the serum-free 2i medium supplemented with MEK/ERK pathway and GSK3 inhibitors and 3i medium additionally containing a FGF receptor inhibitor (Buehr et al. 2008; Li et al. 2008) Kawamata and Ochiya used the serum-containing YPAC medium furthermore comprising chemical inhibitors for the TGF-β receptor Alk5 and rho kinase inhibitor (ROCKi) for rat ESC culture (Kawamata and Ochiya 2010). In contrast to feeder-free conditions developed for pluripotent mouse ESC *in vitro* culture a feeder layer seemed to be essential for rat ESCs, embryonic germ cells (EGC) and induced pluripotent stem cells (iPS) (Furue et al. 2005; Nichols and Ying 2006; Blair et al. 2011a; Northrup et al. 2011). In this study we showed that the immortalized tumor rat fibroblast cell line TRF-O3 as innovative feeder cells supported the culture of rat pluripotent and germ-line transmissible ESCs. Usage of TRF-O3 feeder cells was a time saving, cost-effective approach to minimize animal usage by avoiding the repeated preparation of fresh embryonic fibroblasts.

The first mouse ESCs were cultured in 1984 from the 129/*ter*Sv strain carrying a point mutation in the *Dnd1* gene referred to as *Ter* (Stevens 1973; Wobus et al. 1984). ESCs derived from the 129SV background became subsequently a favored tool in mouse gene targeting experiments (Seong et al. 2004; Blair et al. 2011a). We described a similar mutation in the rat *Dnd1* gene of the WKY/Ztm strain, which was therefore denominated *ter* (Northrup et al. 2012) leading to the hypothesis that the WKY strain might be the superior genetic background for the cultivation of rat ESCs. In this work we figured out that the WKY/Ztm strain as a favored genetic background facilitates the efficient derivation of male and female ESCs together with improved culture conditions using 2i-LIF medium and TRF-O3 feeder cells.

## Materials and methods

### Animals

Rats and mice were bred and maintained at the Central Animal Facility of the Hannover Medical School, Carl-Neuberg-Strasse 1, 30625 Hannover, Germany (subline code: Ztm: http://www.mhhannover.de/ztl.html). The experiments were in accordance with the German Animal Welfare Legislation (Tierschutzgesetz in der Fassung 2006). They were approved by the local Institutional Animal Care and Research Advisory Committee, and permitted by the Animal Welfare Service of the Lower Saxony State Office for Consumer Protection and Food Safety (Az.09/1773).

### Husbandry

WKY/Ztm, WKY-*Dnd1*^*ter*^/Ztm rats and NMRI mice were maintained on sterilized softwood granulate bedding (Lignocel, Altromin, Lage, Germany) at a temperature of 22 ± 2°C and relative humidity of approximately 55 ± 5% under a 14:10 h light–dark cycle They received an autoclaved commercial pelleted diet (Altromin 1314) (protein 22%, fat 5%, raw fiber 4.5%, ash 7%, utilizing energy 3.1 kcal/g) and water *ad libitum*. Mice and rats were monitored for microorganisms according to the FELASA recommendations (Nicklas et al. 2002). In addition the WKY-*Dnd1*^*ter*^/Ztm rats were serologically negative for Hanta, Kilham rat, PVM, Reo3, Sendai, SDA, rat corona, Theiler´s encephalomyelitis, and Toolan´s (H1) viruses. The colony was maintained as a segregating inbred strain by mating littermates or parents known to carry the mutation. The rats were caged in groups of three animals, in type IVs Macrolon cages (1370 cm^2^).

### Isolation and culture of murine Fibroblasts

NMRI mouse embryos were collected at the 14th day post coitum (dpc), WKY/Ztm rat embryos on 15 dpc. Testicular and ovarial teratomas derived from *Dnd1*-deficient WKY/Ztm rats were prepared at 6 weeks of age. The embryos and tumor tissues were chopped to 2–3 mm pieces and stirred with 0,05% trypsin (Biochrom, Berlin, Germany) for 4 hours at 37°C. Every 30 minutes the tissue lysate was transferred to a fresh tube and centrifuged at 1200 rpm at 4°C for 10 min. The cell pellets were then resuspended in culture medium and stored on ice. Finally the different fibroblast fractions were pooled and the cells were seeded at 10^6^ cell/ml in cell culture flasks. Mouse embryonic fibroblasts (MEF), rat embryonic fibroblasts (REF), and tumor derived rat fibroblasts (TRF) were split every 3–4 days using trypsin and feeder cell medium containing DMEM (Biochrom), 15% FCS superior (Biochrom), 4 mM L-glutamine (Biochrom), 100 μg/ml penicillin/streptomycin (Biochrom), 1 mM sodium pyruvate (Biochrom) and 1× NEAA (Sigma-Aldrich, Munich, Germany). NIH/3 T3 cells (ATCC No: CRL-1658) were maintained according to the supplier recommendations.

### Fibroblast specific gene expression

RT-PCR analysis were performed to determine the expression levels of the fibroblast-specific genes *collagen alpha-2(I) chain (Col1a2), vimentin, prolyl 4-hydroxylase, alpha polypeptide II (P4ha2),* S100A4, and smooth muscle α-actin *(Acta2)* in mouse and rat fibroblasts. The transcripts of the secreted factors *basic fibroblast growth factor (FGF2), bone morphogenetic protein 4 (BMP4), leukemia inhibitory factor (LIF)* and *stem cell factor (SCF)* were also amplified together with *GAPDH* as endogenous control. Primer sequences were summarized in Additional file 1: Table S1.

### Feeder cells preparation

Cell lines derived from the murine and human female reproductive tract ED27 (trophoblast), Rcho-1 (chorioncarcinoma), RENTRO1 (endometrium) and the lines OE-E6/E7, BM1.11 as well as BM12.4 (oviductal epithelium) were cultured as described earlier (Derbigny et al. 2005, Kniss et al. 2001, Lee et al. 2001, Sahgal et al. 2006, Wiehle et al. 1990) (Additional file 1: Table S1). For feeder layer production cells were washed 3× with PBS and detached with 0,25% trypsin at 37°C for 10 minutes and centrifuged at 1200 rpm at 4°C for 5 min. After γ-irradiated with 50 Gy (Gammacell 2000, Molsgaard Medical, Copenhagen, Denmark) the mitotic inactivated cells were seeded with 10^6^/ml in feeder cell medium to achieve a confluent layer.

### Cultivation of rat embryonic stem cells

The reproductive cycle of eight weeks old female WKY/Ztm rats was synchronized through a subcutaneous injection of 50 μg luteinizing-hormone-releasing hormone (LHRH) (Sigma-Aldrich) four days before mating. Sperm positive females were determined by vaginal next morning. Inseminated females were sacrificed at 5 dpc and blastocysts were flushed out of the uteri using 3 ml modified M2 medium. Blastocysts were transferred to Tyrode’s solution (Nagy et al. 2003) to remove the Zona pelucida, and washed subsequently four times in modified M2 medium. Hatched blastocysts were then stored in modified M16 medium (Nagy et al. 2003) containing 1% NEAA (Sigma-Aldrich) and incubated with a 1:10 dilution of rabbit anti-rat serum (Hogan et al. 1994, Section D) for 30 minutes at 37°C for immunosurgery (Solter and Knowles 1975, Spielmann et al. 1980, Nagy et al. 2003). Blastocysts were washed three times in modified M16 medium followed by 30 minutes incubation in a 1:8 dilution of guinea pig complement (Sigma-Aldrich) and additional three washing steps in modified M16 medium. Then the ICMs were dissociated from the trophoblast cells by pipetting, and cultured in 2i with 1000 units rat LIF/ml (Northrup et al. 2011), YPAC medium (Kawamata and Ochiya 2010) or 4i (YPAC without serum supplemented with 1000 units/ml rat LIF) on mitotic inactivated TRF-O3 as feeder cells (Northrup et al. 2011).

### Blastocyst injection and embryo transfer

Blastocyst injection was performed as described earlier with some modifications. (Hogan et al. 1994) DA.1 M blastocysts were injected at 5 dpc. 10 embryos were transferred to each uterine horn of a pseudopregnant rat. Chimeric rats were identified by coat color. Germline transmission was tested by mating of chimeras with WKY/Ztm rats (Hedrich 1990).

### RNA preparation and reverse transcription

Fibroblasts were harvested by scrapping, while the loosely attached colonies of ESCs were detached by pipetting leaving behind the feeder cells. RNA was isolated using Qiashredder and the RNeasy Mini Kit (Qiagen, Hilden, Germany) in accordance to supplier’s recommendations. cDNA synthesis was performed using the Omniscript Reverse Transcription Kit (Qiagen) with a mix of Oligo(dT) and Random Hexamer Primers (Fermentas, St. Leon-Rot, Germany) following the manufacturer’s instructions.

### Characterization of rat embryonic stem cells

The PCR amplification of stem cell specific genes and for the germ layer specific markers *T-brachyury* (mesoderm), *nestin* (ectoderm) and *α-fetoprotein* (entoderm) were described earlier (Winnier et al. 1995; Li et al. 2008; Buehr et al. 2008). Determination of rat *c-kit* and mouse *GAPDH* transcription were performed as previously described (Robbinson and McKinney 1992, Miyamoto et al. 2010). Alkaline phosphatase staining, chromosome count and immunocytofluorescent stainings were done as described earlier using anti OCT4 (1:25; ab18976; Abcam, Cambridge, UK), anti NANOG (1:25; RCAB002P-F; ReproCell, London, UK), anti SSEA-1 (1:25; bs-1702R, Bioss, Woburn, MA) and anti SSEA-3 (1:25; bs-3575R; Bioss) antibodies (Northrup et al. 2011).

### Teratoma Induction

ESC were harvested and resuspended in PBS to form a slightly clumpy suspension. Cells were drawn into a 1 ml syringe, kept at 4°C and warmed to room temperature prior to injection. Teratomas were then induced by subcutaneous injection of the 5×10^6^ ESCs in 150 μl of PBS cells into the ventro-lateral region of NOD.CB17-*Prkdc*^*scid*^/J mice. Teratomas were removed when they had reached a maximal diameter of 1–1.5 cm or weight loss of over 20% was evident in the recipients. Formalin-fixed and paraffin-embedded teratomas were cut into 4 μm slices before staining with hematoxillin/eosin. Microphotographs were taken using a Zeiss AxioCam MRc camera and analyzed histologically for structures of all three germ layers.

## Results

### Generation of fibroblast cell lines of mouse and rat origin

**Figure 1:**
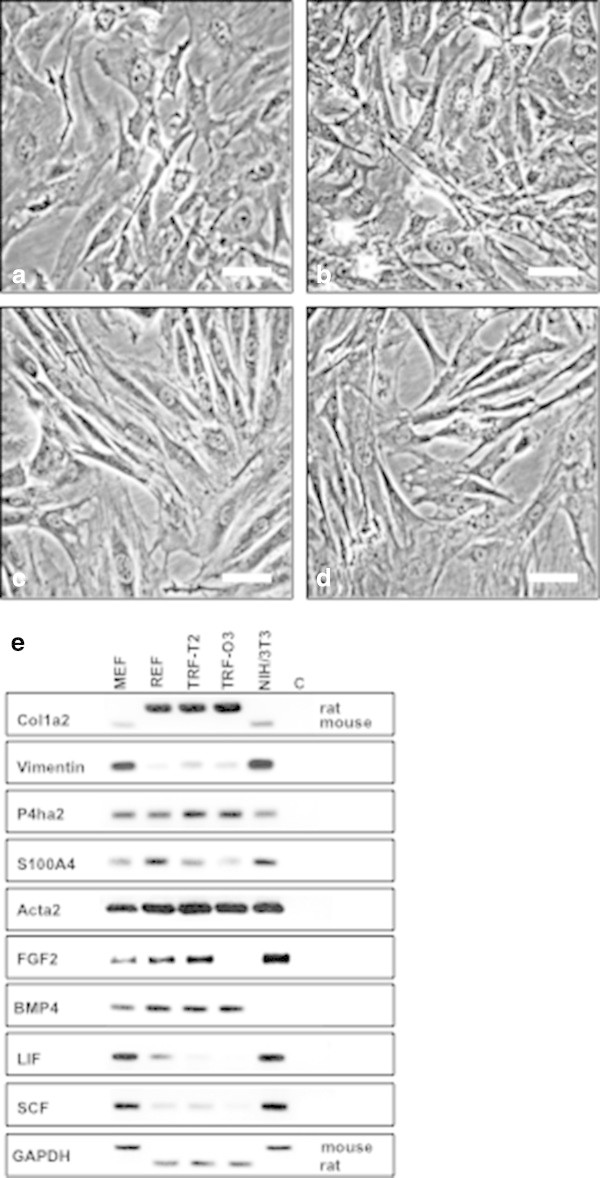
**Characterization of murine fibroblasts.** Phase contrast images (20×) of **a**. rat embryonic fibroblasts (REF), **b**. mouse embryonic fibroblasts (MEF), **c**. female tumor rat fibroblasts (TRF-O3) and **d**. male tumor rat fibroblasts (TRF-T2) **e**. RT-PCR analysis of the expression patterns of fibroblast specific genes and important paracrine factors. GAPDH was used as endogenous control.

Growth factors, cytokines, and extracellular matrix components secreted from feeder cells contribute to the maintenance of ESC pluripotency *in vitro*. Therefore, we cultured and characterized REFs derived from the strain WKY/Ztm as well as MEFs from the NMRI strain. Furthermore, TRF cell lines were isolated from male and female teratomas caused by *Dnd1* deficiency in WKY/Ztm rats. Morphological evaluation of the cells *in vitro* confirmed a polygonal shape with long filopodia typical for embryonic fibroblasts from rat (Figure 1a) and mouse (Figure 1b) while the female TRF-O3 (Figure 1c) and male TRF-T2 (Figure 1d) cells were more spindle-shaped and elongated forming a dense confluent cell layer. TRFs were maintained for more than 50 passages without morphological signs of senescence or reduced proliferation capacity in contrast to rapid loss of viability in MEFs. To differentiate between normal fibroblast and carcinoma-associated fibroblasts (CAF) the expression of fibroblast specific genes, and of *Acta2* as marker for myofibroblast were performed (Egeblad et al. 2005; Kalluri and Zeisberg 2006). RT-PCR analysis revealed high expression of *Col1a2* in REFs, TRF-O3, and TRF-T2 cells but showed only sparse *Col1a2* mRNAs in the murine cell lines. Reciprocally, *vimentin* showed much higher transcription rates in MEFs and NIH/3 T3 cells derived from mouse embryos in contrast to the weak activity in rat fibroblasts (Figure 1e). As expected, the key enzyme of the collagen synthesis process *P4ha2* was detected at the same transcription level in all fibroblast cell lines tested, while slight expression of the *S100A4* gene (also known as fibroblast specific protein 1 in mouse and human) was found in MEFs and TRFs. Only REFs and NIH/3 T3 cells showed higher transcription rates of *S100A4. Acta2* was expressed equally in all cell lines investigated. The paracrine factors *LIF* and *SCF* were highly transcribed in MEFs and NIH/3 T3 cells but not in the rat cells while inversely the transcription of *BMP4* was significantly higher in rat fibroblasts than in MEFs or NIH/3 T3 cells (Figure 1e). These fibroblast cell lines were used in comparison to epithelial cell lines derived from female genital tract of mouse, rat and human as well as chorion carcinoma cells to identify the optimal feeder cell type that provides culture conditions supporting the pluripotency of ESCs *in vivo*.

### Determination of the optimal feeder cell line

After immunosurgery inner cell masses derived from wild type WKY-*Dnd1*^*ter*^/Ztm rats were seeded on oviductal epithelial cells of human and mouse origin, rat endometrial cell lines, human trophoblast stem cells and rat chorioncarcinoma cell (Additional file 2: Table S2) as well as on MEFs, REFs and TRFs. After one week of culture in 2i-LIF medium clonal growth of ESCs as spherical colonies with a smooth surface and sharp refractive edges was only observed on TRF-O3 as feeder cells while on TRF-T2 a lot of growing ES cell clones started to differentiate. Clonal growth of pluripotent ES cell was absent using the other cell lines as feeders (data not shown). Therefore, TRF-O3 efficiently supports the growth and maintenance of pluripotent rat ESCs in 2i-LIF medium and was used in all further experiments.

### Establishment and characterization of rat embryonic stem cell lines

**Figure 2:**
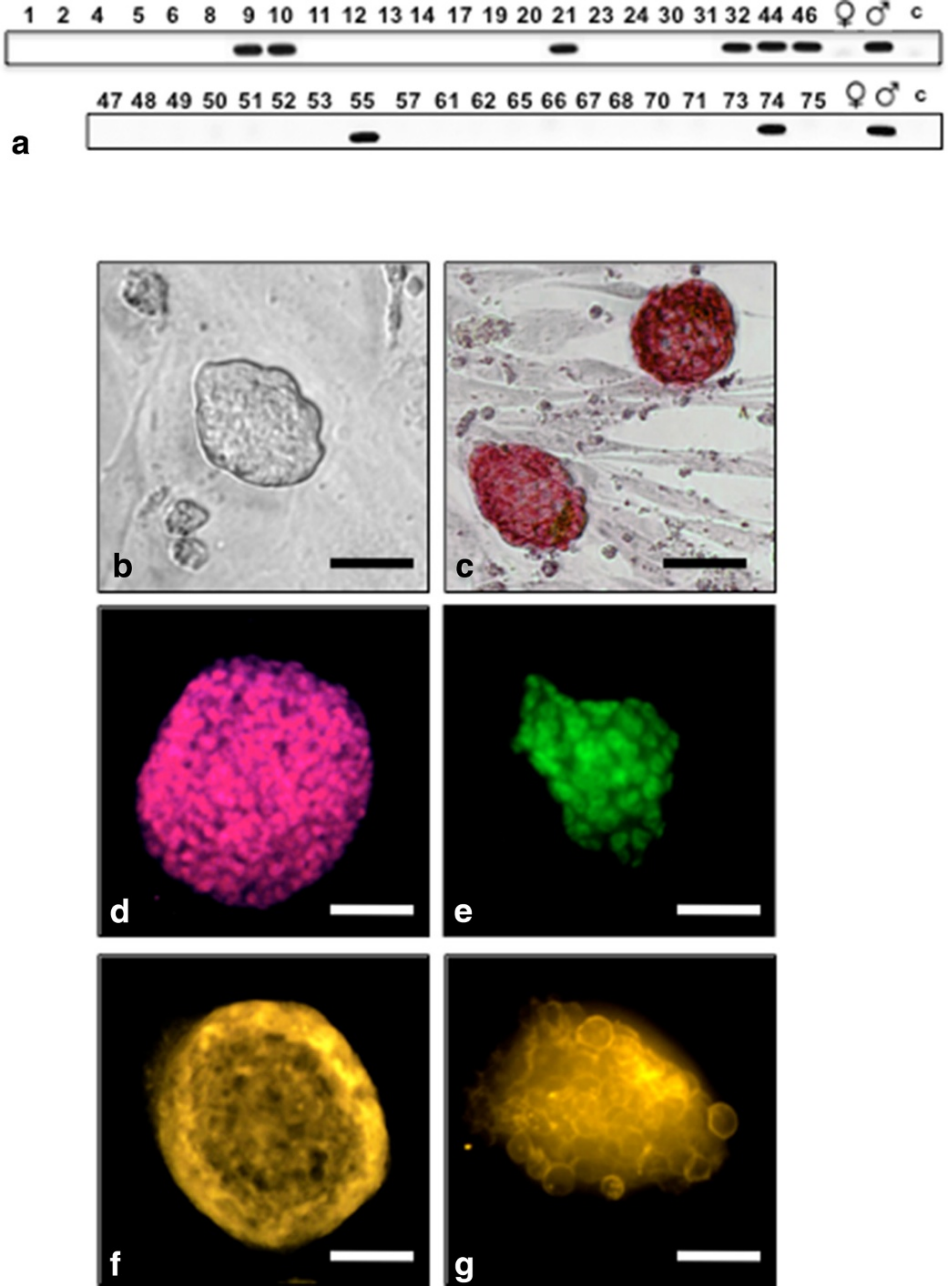
**Gender determination of pluripotency rat ES cells. a**. SRY-PCR demonstrated that 8 male ESC lines out of 43 were established in two separate experiments. The rat ESC line ES21 forms undifferentiated pluripotent colonies demonstrated by **b**. phase contrast, **c**. alkaline phosphates expression, and positive stainings for **d**. Nanog, **e**. Oct4, **f**. SSEA-1 and **g**. SSEA-3 (10×); scale bar is 100 μm.

ICMs from 4.5 dpc blastocysts were isolated through immunosurgery and seeded on mitotic-inactivated TRF-O3 cells as feeders in 2i-LIF medium on 96 well plates in two different experiments. In the first round 23 ICMs out of 46 showed clonal growth representing a 50% plating efficiency. 6 of these established ESC lines were identified as male (26.1%). A plating efficiency of 71% was achieved in the second round with 20 ESC lines derived from 28 ICMs containing 2 male ESC lines (10.0%) (Figure 2a). The ESC clones appeared to be round-shaped colonies with smooth surface and sharp edge (Figure 2b) consisting of alkaline phosphatase positive cells (Figure 2c). Immunocytofluorescent staining revealed the expression of the pluripotency markers *SSEA-1, SSEA-3* (Northrup et al. 2011; Fernandez et al. 2011)*, Nanog and Oct4* (Figure 2d- 2g) as well as the primordial germ cells - specific gene DDX4/MVH (data not shown) in the ESC lines.

**Figure 3:**
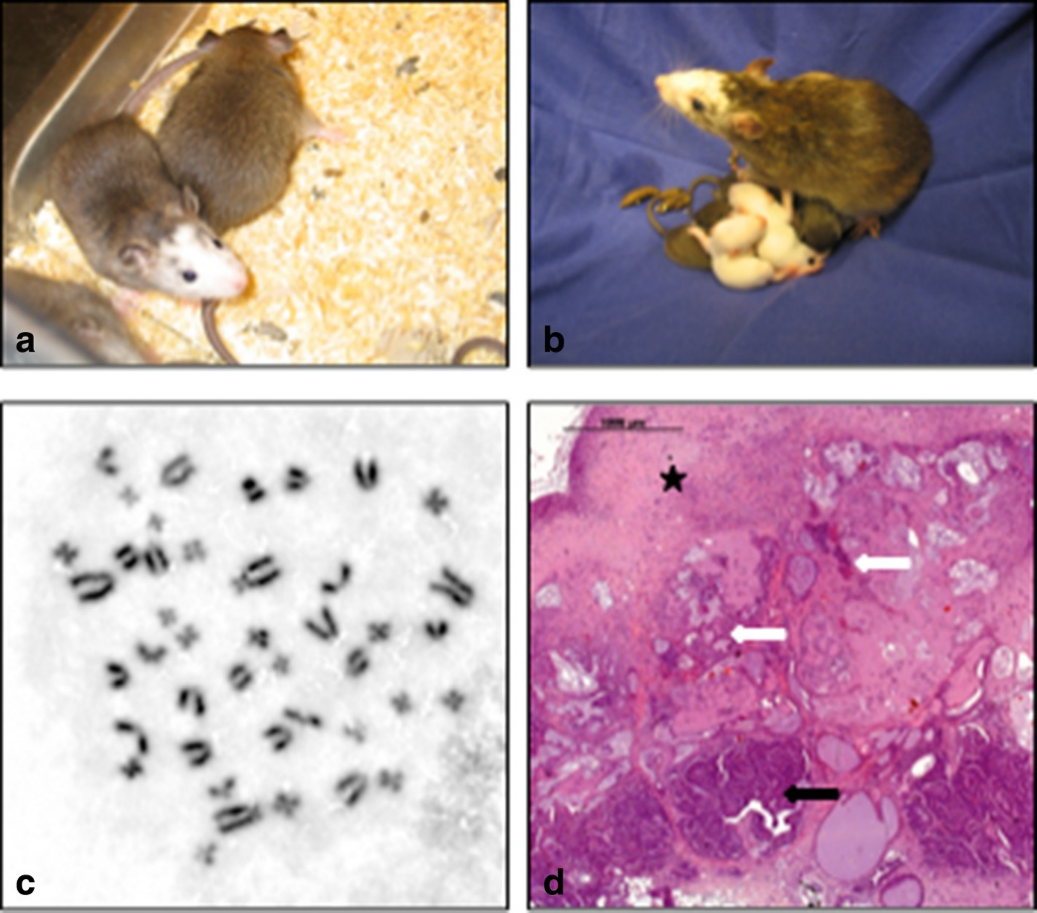
**Analysis of ESC line ES21. a**. ES21 cells contributed to chimeric rats after blastocyst injection and **b**. showed germ line transmission. **c**. Metaphase spreading and giemsa staining proofed that ES21 cells at passage 12 were genetically stable containing a diploid set of chromosomes. **d**. Teratomas with tridermal tissues (white arrow: glandular endodermal structures; black star: muscle-like mesodermal tissue; black arrow: ectodermal neural tube formation) arose from ES21 cells after subcutaneous injection into immuno-deficient mice, H&E, (2.5×).

**Figure 4:**
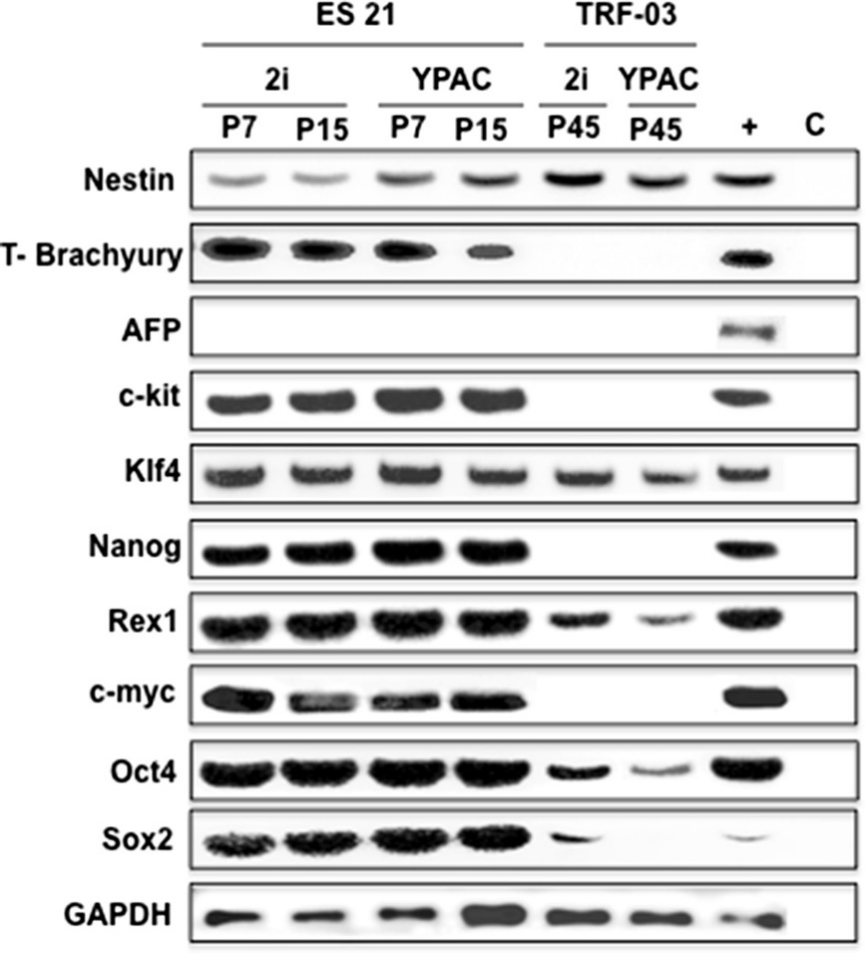
**Expression patterns of ESC compared to TRF-O3 feeder cells in 2i and YPAC medium.** Transcription levels of pluripotency and differentiation markers were compared in ES21 from passage 7 and passage 15 cultured of TRF-O3 as feeder cells in 2i-LIF and YPAC medium performing RT-PCR. **+** positive control: pooled cDNAs from pluripotent rat embryonic germ cell lines (Northrup et al. 2011). GAPDH was used as endogenous control.

Only the microinjection in blastocysts of the female cell lines ES1 and ES10 and male line ES21 resulted in chimeric rats, while ES9 cells did not integrate into the embryos. Two highly chimeric females (out of 3 male and 2 female chimeric offspring) (Figure 3a) that originated from ES21 showed germ line transmission (Figure 3b). Therefore, the ESC line ES21 was analyzed in more detail. Chromosome counting revealed a diploid karyotype of ES21 in more than 80% of the metaphases (Figure 3c). Subcutaneous injection of ES21 cells into immunodeficient mice led to the formation of teratomas containing tissues derived from all three germ layers demonstrating pluripotency (Figure 3d). The expression pattern of ES21 was characterized through the transcription of the pluripotency markers *c-kit, Klf4, Nanog, Rex1, Oct4* and *Sox2* as well as the proto-oncogene c-myc (Figure 4). In contrast to the ESCs the underlying feeder cells showed weak expressions of *Rex1, Oct4* and *Sox2*. Only the *Klf4* activity was on the same level in the tumor rat fibroblasts and in the ES cells (Figure 4). Moreover, RT-PCR amplification revealed no expression of the entodermal marker *AFP*, a slight transcription of *nestin* as an early ectoderm marker, and an elevated expression of *T-Brachyury* as a marker for the early mesodermal cell lineage in ES21 cells (Figure 4).

**Figure 5:**
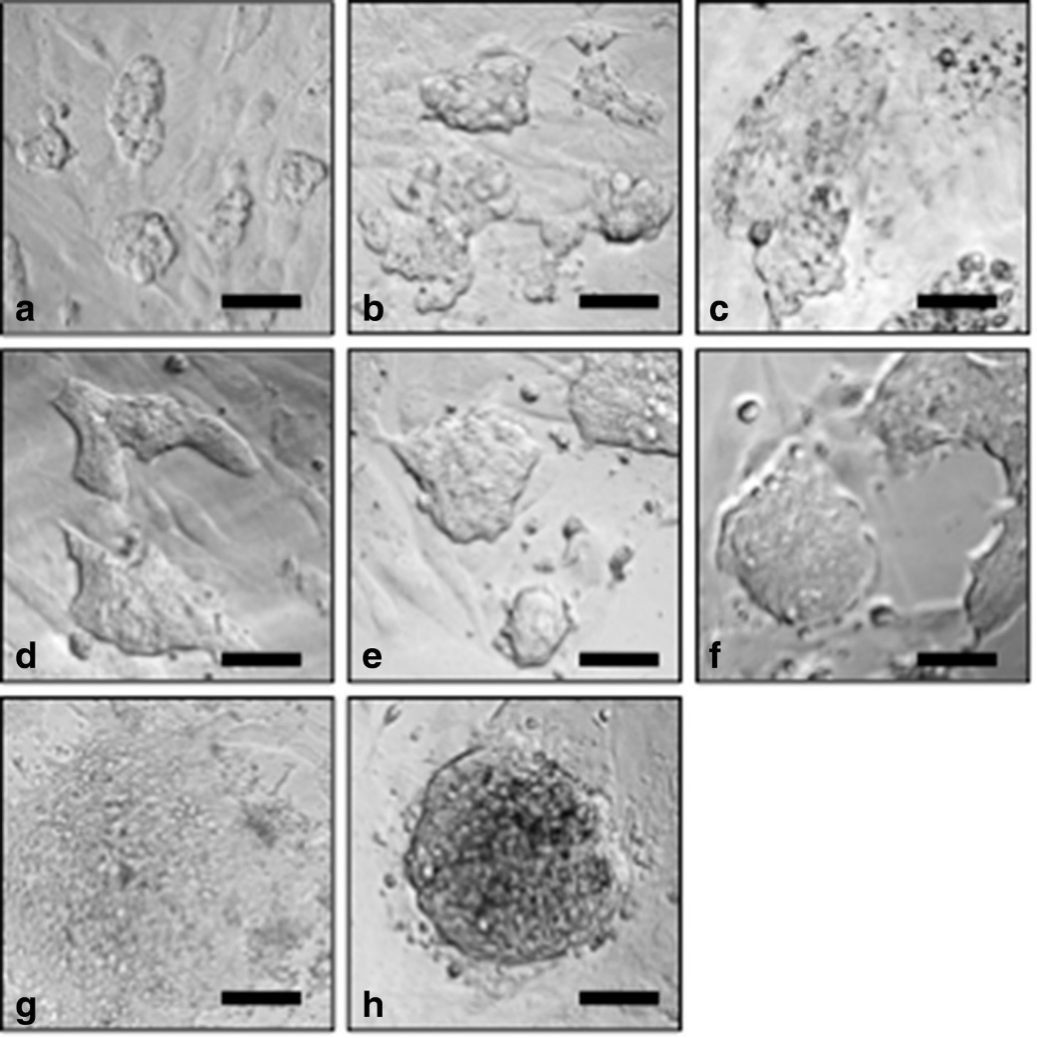
**Differentiation of ES21 cells and primary ESC in YPAC or 4i medium on TRF-O3 cells. a**-**c**. Differentiation of ES21 cells started between day 1 and day 3 after cultivation in YPAC medium. **d**-**f**. In contrast the ESC remained undifferentiated when synchronously cultured in 2i-LIF medium. Freshly isolated ESC also underwent differentiation in **g**. YPAC medium or **h**. 4i medium, phase contrast (10×), scale bar is 100 μm.

ES21 cells were also transferred to YPAC medium supplemented with TGF-β receptor Alk 1 inhibitor, ROCKi and fetal bovine serum (Kawamata and Ochiya 2010) to achieve a potential improvement in the maintenance of stemness and proliferation of ESCs compared to the culture in 2i-LIF medium. Unfortunately, the cultivation of ES21 cells in YPAC medium led to a higher ratio of ES cell colonies with morphological differentiation (Figure 5a, b and c) in contrast to culture in 2i-LIF medium (Figure 5d, e and f). Moreover, the differentiation process of ES21 cell in YPAC was associated with an increase of expression of the early ectodermal marker *nestin* and a reduced transcription of the endodermal marker *T-Brachyury* (Figure 4) suggesting that YPAC enhanced the development of ES21 into the ectodermal progenitor cell lineage. Also freshly isolated ICMs were seeded onto TRF-O3 feeder cells in YPAC medium to exclude that the differentiation of ES21 cells in YPAC medium was due to selection of 2i-LIF medium conditioned ES21 cells. But only rough colonies consisting of diverse cell types emerged from freshly prepared ICMs in YPAC medium (Figure 5g) as well as in 4i medium (YPAC without serum) demonstrating that the interaction of TRF-O3 feeder cells with the TGF-β receptor inhibitor or the ROCKi might initiate differentiation of ES21 cells under these conditions (Figure 5h).

## Discussion

Feeder cells provide extracellular matrix components, growth factors and cytokines supporting the cultivation of pluripotent stem cells from mouse and man (Nagy et al. 2003, Trounson 2006; De Felici et al. 2009; Blair et al. 2011a; Lam and Longaker 2012). Also rat ESCs are cultured on genetically engineered MEFs after mitotic inactivation. DIA-M cells expressing LIF after stable transfection in C3H-MEFs served as feeder for cultivation of the first pluripotent rat ESCs in 2i-LIF medium (Rathjen et al. 1990; Buehr et al. 2008). Concurrently, Li and colleagues used L cells (adult murine fibroblasts derived from C3H/An mice) mixed with MEFs or DR4 cells (MEF resistant to neomycin, hygromycin, puromycin and 6-thioguanin) for derivation of rat ESCs in the same medium (Li et al. 2008). Moreover, rat ESC, pluripotent embryonic germ cells and iPS cells are maintained on naive MEFs derived from different mouse strains (Liao et al. 2009; Li et al. 2009b; Hirabayashi et al. 2010; Kawamata and Ochiya 2010; Zhao et al. 2010; Fernandez et al. 2011; Yamamoto et al. 2011). In this work we demonstrated that the tumor derived rat fibroblast cell line TRF-O3 served as innovative feeder layer for the enhanced culture of rat pluripotent and germ-line transmissible ESCs. Beside the characteristic fibroblast-like shape RT-PCR analysis revealed strong expression of the fibroblast-specific markers *Col1a2* and *P4ha2* (Kalluri and Zeisberg 2006) essential for the biosynthesis of collagens as fibrillar extracellular matrix components in TRF-O3 cells. In contrast, the intermediate-filament protein *Vimentin* commonly expressed in normal fibroblast and the *fibroblast specific protein 1 (FSP1*)-homolog S100A4 (Strutz et al. 1995; Wang and Stamenovic 2002; Helfand et al. 2004; Kalluri and Zeisberg 2006) were only weakly transcribed in TRF-O3 cells. Reduction of *vimentin* expression was also found in REFs immortalized through overexpression of the human mitochondrial ribosomal protein MRPS18-2 and was associated with the loss of Acta2 protein in comparison to untreated control REFs (Yenamandra et al. 2012). On the other hand Sugimoto and coworkers identified a class of CAFs in the mouse highly positive for *Acta2* in combination with reduced *S100A4* expression (Sugimoto et al. 2006). Moreover, *nestin* expression in TRF-O3 cells correlates with the detection of *nestin* mRNA in human myofibroblasts and in scar myofibroblasts isolated from post-infarcted rat myocard (Béguin et al. 2011; Kishaba et al. 2010). Therefore, high *nestin* and *Acta2* transcription rates together with the low expression of *vimentin* and *S100A4* characterized TRF-O3 cells as activated fibroblasts or myofibroblasts (Egeblad et al. 2005; Kalluri and Zeisberg 2006; Grigorian et al. 2008). Additionally, RT-PCR analysis of TRF-O3 cells revealed strong expression of the paracrine factor *BMP4* while *FGF2, LIF* and *SCF* transcription were absent. The growth factor LIF is an indispensable component essential for the maintenance of pluripotent ESCs derived from mice and rats (Nagy et al. 2003, Buehr et al. 2008; Li et al. 2008; Blair et al. 2011a, Hong et al. 2012). Only slight *LIF* expression was detected in REFs derived from 14.5 dpc embryos while in TRFs originating from 6 weeks old rats *LIF* expression was absent. These results are in line with the age-dependent silencing process found by Takahama and co-workers demonstrating the loss of *LIF* expression in REFs between day 10 and 15 dpc (Takahama et al. 1998). Therefore, 2i medium supplemented with a higher concentration of recombinant rat LIF was necessary for the cultivation of rat ESCs in combination with TRF-O3 feeder cells in this work (Buehr et al. 2008; Li et al. 2008). This was in consistency with the optimal culture conditions for pluripotent EGCs as a closely related, DDX4/MVH positive stem cell types (Leitch et al. 2010; Northrup et al. 2011).

BMP4, SCF and bFGF are involved in the regulation of proliferation and survival as well as in the maintenance of pluripotency of mouse ESC and EGC *in vitro* (reviewed in De Felici et al. 2009). Two workgroups showed in 2008 that the 3i medium containing the FGF2 inhibitor SU5402 enabled the first successful generation of rat ESC (Li et al. 2008; Buehr et al. 2008). But 2i-LIF medium containing only glycogen synthase kinase 3 (GSK3) and MEK/ERK pathway inhibitors were also sufficient for rat ESC derivation emphasizing that the cytokines FGF2 and SCF are dispensable rat ESC culture (Bashamboo et al. 2006; Leitch et al. 2010). Considering these results the lack of *bFGF* and *SCF* expression was one of the beneficial aspects using TRF-O3 as feeder cells for the cultivation of rat stem cells.

On the other hand the *BMP4* expression in TRF-O3 cells correlated with the induction of *T-brachyury* and *nestin* transcription as marker genes for the differentiation of mesodermal and ectodermal precursor cells in the ESC population. Winnier and colleagues demonstrated that BMP4 induces mesoderm formation in the early mouse development (Winnier et al. 1995). *In vitro* BMP4 facilitates the differentiation of mouse ESCs into mesodermal precursors associated with a weak induction of *nestin* expression (Torres et al. 2012). The latter result showed that BMP4 also triggered the differentiation of mouse ESCs into the ectodermal cell lineage and confirmed the development of ectodermal lineage cells in embryoid bodies after 4 and 6 days cultured with BMP4 (Harvey et al. 2010). Despite the tendency to differentiate into the mesodermal and ectodermal cell lineage culture of ICMs on TRF-O3 feeder cells with 2i-LIF medium resulted in pluripotent, genetically stable and germ-line transmissible rat ESCs. To further improve the culture conditions we tested the YPAC medium (Kawamata and Ochiya 2010; 2011) containing additional TGF-β and ROCKi and 4i medium (YPAC without serum) together with the TRF-O3 feeder cells. This combination enhanced differentiation of rat ES21 colonies as well as freshly seeded ICMs with increased *nestin* expression demonstrating accelerated transformation to ectodermal cells. In human ESC and iPS cell lines ROCKi increased proliferation, blocks apoptosis, promotes survival and improves plating efficiency of pluripotent stem cells (Pakzad et al. 2010; Gauthaman et al. 2010; Olmer et al., 2010). No such positive effects of ROCKi on rat ESCs *in vitro* were observed using TRF-O3 feeder cells in this study.

While several approaches to generate ESCs originated from DA, Fischer344, and Wistar inbred strains as well as from the SD outbred strain led mainly to female ESC lines (Li et al. 2008; Kawamata and Ochiya 2010; Blair et al. 2011a; Fernandez et al. 2011) Hong and coworkers established 12 male and 8 female ESC lines from DA or Fischer344 embryos under varying culture conditions (Hong, et al. 2012). Buehr and colleagues also established 5 male ESC lines out of 13 from Fischer344 rats and even 14 out of 25 ESC lines derived from DA blastocysts (Buehr et al. 2008). We demonstrated in this work that the cultivation of WKY/Ztm ICMs in 2i-LIF medium on TRF-O3 feeder cells resulted reproducible in up to 26% male ESC lines. Moreover, the same phenomenon was observed culturing genital-ridge derived EGC derived from 14.5 dpc embryos with 3 male out of 11 cell lines as well as from pre-migratory EGCs from day 10.5 dpc embryos with 8 male out of 21 cell lines using the WKY-*Dnd1*^*ter*^*/Ztm* strain (Northrup et al. 2011). The significant selection advantage of female stem cell lines was likely due to the slower proliferation of male cells in early passages, an observation also described by Blair and colleagues (Blair et al. 2011b).

Therefore, TRF-O3 as feeder cells represents an alternative tool for the generation of pluripotent stem cells from rats using 2i-LIF medium. Derivation of rat stem cells with WKY/Ztm background resulted under these conditions in a significant higher rate of male cell lines than from most other inbred strains and cell culture systems so far tested. Introduction of resistant gene expression cassettes will further improve the versatility of TRF-O3 cells for the establishment of genetically manipulated rats.

## Electronic supplementary material

Additional file 1: **Primers for the gene expression analysis of the feeder cell lines.** (DOCX 99 KB)

Additional file 2: **Cell lines tested as feeder cells for rat ES cell culture.** (DOCX 56 KB)
