# Supplementary material for: Immortalized tumor derived rat fibroblasts as feeder cells facilitate the cultivation of male embryonic stem cells from the rat strain WKY/Ztm
Source: Springerplus. 2014 Oct 8;3:588. doi: 10.1186/2193-1801-3-588 (PMC4197200; doi:10.1186/2193-1801-3-588)
Supplement: Supplementary file 1 — Additional file 1: Primers for the gene expression analysis of the feeder cell lines. (DOCX 99 KB) [file 40064_2014_1293_MOESM1_ESM.docx]

*Supplementary Table 1*

*Rattus norvegicus* product

Acta2: forward: 5’-AGGGCTGTTTTCCCATCCATC-‘3 206bp

reverse: 5’-CCACGCGTTGCTCGTTATAGA-‘3

BMP4: forward: 5’-AGCCAACACTGTGAGGAGTTTC-‘3 78bp

reverse: 5’-ACGAAAAGCAGAGCTCTCACTG-‘3

Col1a2 forward: 5’-CAAATGGGATCTGTACGCAAGG-‘3 196 bp

reverse: 5’-CCTTTGTCAGAATACTGAGCAGC-‘3

FGF2 forward: 5’-GTGTCTATCAAGGGAGTGTG-‘3 240 bp

reverse: 5’-TCAGTGCCACATACCAACTG-‘3

FSP1 forward: 5’-ACAGAGCTCAAGGAGCTACTGA-‘3 84 bp

reverse: 5’-TCATCAGCTTCTGGAATGCAGC-‘3

LIF forward: 5’-AACTCAACGGCAGTGCCAATGC-‘3 61bp

reverse: 5’-AAATGGTTCCCCTTGAGCTGTG-‘3

P4ha2 forward: 5’-GAAGACTCAGAAAGTCCGTGTC-‘3 67 bp

reverse: 5’-CCTGGAGCTTCATGACTGGAAA-‘3

SCF forward: 5’-GCGCTGCCTTTCCTTATGAAGA-‘3 104bp

reverse: 5’-CCTGCAGATCTCCTGAGTTTTG-‘3

Vimentin forward: 5’-GTGGATGCCCTTAAAGGCACTA-‘3 68 bp

reverse: 5’-TCTTCCATTTCACGCATCTGGC-‘3

*Mus musculus spec*. product

Acta2: forward: 5’-AGGGCTGTTTTCCCATCCATC-‘3 206bp

reverse: 5’-CCACGCGTTGCTCGTTATAGA-‘3

BMP4: forward: 5’-AGCCAACACTGTGAGGA-‘3 78bp

reverse: 5’-ACGAAAAGCAGAGCTCT-‘3

Col1a2 forward: 5’-AGATGGTGTTGATGGTCC-‘3 120 bp

reverse: 5’-CCTTTGTCAGAATACTGA-‘3

FGF2 forward: 5’-GTGTCTATCAAGGGAGTGTG-‘3 240 bp

reverse: 5’-TCAGTGCCACATACCAACTG-‘3

FSP1 forward: 5’-ACAGAGCTCAAGGAGCT-‘3 84 bp

reverse: 5’-TCATCACCTTCTGGAATG-‘3

LIF forward: 5’-AGCTCAATGGCAGCGCC-‘3 61bp

reverse: 5’-AAACGGCTCCCCTTGAG-‘3

P4ha2 forward: 5’-GAAGACTCAGAAAGTTCA-‘3 67 bp

reverse: 5’-CCTGGAGCTTCATAACTG-‘3

SCF forward: 5’-GCGCTGCCTTTCCTTATG-‘3 104bp

reverse: 5’-CCCGCAGATCTCCTTGGT -‘3

Vimentin forward: 5’-GTGGATGCCCTTAAAGG-‘3 63 bp

reverse: 5’-TCTTCCATCTCACGCATC-‘3
