# Supplementary material for: Immortalized tumor derived rat fibroblasts as feeder cells facilitate the cultivation of male embryonic stem cells from the rat strain WKY/Ztm
Source: Springerplus. 2014 Oct 8;3:588. doi: 10.1186/2193-1801-3-588 (PMC4197200; doi:10.1186/2193-1801-3-588)
Supplement: Supplementary file 2 — Additional file 2: Cell lines tested as feeder cells for rat ES cell culture. (DOCX 56 KB) [file 40064_2014_1293_MOESM2_ESM.docx]

*Supplementary Table 2*

| **symbol** | **cell line** | **species** | **depositor** |
| --- | --- | --- | --- |
| ED27 | trophoblast cell line | human | D.A. Kniss/ T. Summerfield; Ohio State University |
| Rcho-1 | chorioncarcinoma cell line | rat | M. J. Soares; Kansas City |
| RENTR01 | endometrial cell line; immortalised with SV40 Large T-Antigen and v-Ha-ras | rat | European Collection of Cell Cultures (ECACC) |
| OE-E6/E7 | oviductal epithelial cell line | human | W. S. B. Yeung; University of Hong Kong |
| Bm1.11 | oviductal epithelial cell line | mouse | R. M. Johnson, Indianapolis |
| Bm12.4 | oviductal epithelial cell line | mouse | R. M. Johnson, Indianapolis |
